# Overexpression of ovine *AANAT* and *HIOMT* genes in switchgrass leads to improved growth performance and salt-tolerance

**DOI:** 10.1038/s41598-017-12566-2

**Published:** 2017-09-22

**Authors:** Yan-Hua Huang, Si-Jia Liu, Shan Yuan, Cong Guan, Dan-Yang Tian, Xin Cui, Yun-Wei Zhang, Fu-Yu Yang

**Affiliations:** 1 College of Animal Science and Technology, China Agricultural University, Beijing, China; 2 College of Agronomy and Biotechnology, China Agricultural University, Beijing, China; 3 Beijing Key Laboratory for Grassland Science, China Agricultural University, Beijing, China; 4 National Energy R&D Center for Biomass (NECB), Beijing, China; 5 Beijing Sure Academy of Biosciences, Beijing, China

## Abstract

Melatonin is a well-known bioactive molecule with an array of health-promoting properties. Here, we detected the physiological function of melatonin in transgenic switchgrass overexpressing the homologous sheep arylalkylamine N-acetyltransferase and hydroxyindole *O-*methyltransferase genes, which catalyze the last two steps of melatonin synthesis. Compared to the wild-type (WT) and transgenic control (EV, expressing the empty vector only) plants, the transgenic switchgrass showed higher melatonin levels. Melatonin was detected in almost all switchgrass tissues, and relatively higher levels were detected in the roots and stems. Besides, melatonin showed diurnal or circadian rhythms in switchgrass similar to that in other species. Furthermore, we also found that melatonin positively affected switchgrass growth, flowering and salt tolerance. The genes related to flowering (*APL3*, *SL1*, *FT1*, *FLP3*, *MADS6* and *MADS15*) and salt stress resistance (*PvNHX1*) in transgenic switchgrass exhibited a different expression profiles when compared to the control plants. Our study provided valuable findings that melatonin functions as a promoter in the regulation of switchgrass growth, flowering and salt tolerance.

## Introduction

Melatonin (N-acetyl-5-methoxytryptamine), a well-known indole molecule, has a low molecular weight and simple structure. It occurs ubiquitously in diverse groups of living organisms and performs multiple metabolic functions that influence circadian rhythms^1,2^, reduce oxidative stress^3^, promote embryo implantation^4^, regulate reproduction^2^, and inhibit cancer^5^. Melatonin was first detected in the photosynthesizing alga, *Gonyaulax polyedr* ^6^, and thereafter in several vascular plants^7^. To date, this indoleamine has been widely reported in more than 103 species of monocot and dicot plants^8,9^.

The biosynthetic pathway of melatonin has been comprehensively evaluated early in animals^10^, and recent study reported that mitochondria might be a potential compartment for melatonin synthesis^3^. In the first step of the pathway, tryptophan 5-hydroxylase (T5H) catalyzes the hydroxylation of tryptophan to 5-hydroxytryptophan. The latter is then decarboxylated by aromatic amino acid decarboxylase (AADC), forming serotonin, which serves as the precursor of melatonin. Melatonin synthesis from serotonin is further catalyzed by two enzymes, arylalkylamine N-acetyltransferase (AANAT) and hydroxyindole *O-*methyltransferase (HIOMT). AANAT converts serotonin to N-acetylserotonin and HIOMT serves as the rate-limiting enzyme catalyzing the final synthesis of melatonin from N-acetylserotonin. As in animals, melatonin biosynthesis begins with tryptophan and consists of four-step reactions in plants, but at least six enzymes are involved in this pathway with multiple (at least four) routes^11–13^. Among these enzymes, tryptophan decarboxylase (TDC), tryptamine 5-hydroxylase (T5H), serotonin N-acetyltransferase (SNAT), N-acetylserotonin methyltransferase (ASMT), and caffeic acid O-methyltransferase (COMT), have been characterized at the biochemical and molecular levels, whereas the tryptophan hydroxylase (TPH) has not been studied. Moreover, indoleamine 2,3-dioxygenase (IDO), an enzyme involved in melatonin metabolism in plants, which lowers the intercellular melatonin levels, has also been isolated and characterized^14^. Plants, in contrast to animals, have been found capable of *in vivo* melatonin biosynthesis as well as in incorporating exogenous melatonin from the environment^15^. Therefore, application of the exogenous melatonin can increase the endogenous melatonin levels in plants.

Currently, the physiological functions of melatonin in plants are being intensively explored using either exogenous melatonin treatments or ectopic overexpression of melatonin biosynthetic genes^16^. As a potent free-radical scavenger in animals^3^, melatonin in plants is also believed to function as the first line of defense against adverse environmental conditions imposed by agents like salt, high and low temperatures, drought, UV-B radiation, and heavy metals (see^16–19^ reviews for detailed information). In addition to the ubiquitous function in reactive oxygen species scavenging in both animals and plants, melatonin also plays a role in the regulation of plant growth and development, such as promotion of plant growth and seed germination^20^, protection of plants against senescence^21^, flowering^22^, and stimulation of fruit ripening^23^.

Switchgrass (*Panicum virgatum* L.), a perennial C4 warm-season grass native to North America, has been increasingly recognized as a dedicated bioenergy crop because of its agricultural, industrial, and ecological advantages^24^. Improving the important agricultural traits, such as stress tolerance, is of great significance for switchgrass to adapt to marginal lands, which may compromise plant biomass yield due to adverse environmental stresses. Efforts to increase the salt tolerance through traditional breeding techniques have been challenging because switchgrass is an outcrossing and polyploid species^24^, traits that render it incapable of fulfilling the requirements of large-scale production. Directly introduction of growth and stress-tolerance genes through tissue culture and genetic transformation methods are expected to be more efficient in generating novel switchgrass plants with improved stress tolerance.

Melatonin is a bioactive molecule with important metabolic and defense-related functions, as mentioned above. Several transgenic plants expressing the genes for key enzymes that regulate melatonin synthesis have been demonstrated to improve the tolerance of transgenic plants to unfavorable conditions^16^. Overexpression of human *serotonin N-acetyltransferase* (*SNA*) gene in rice has been reported to improve the cold tolerance of transgenic plants and enhance chlorophyll synthesis in their leaves^25^. Similarly, transgenic tomato overexpressing ovine *AANAT* and ovine *HIOMT* genes exhibited drought resistance^26^. Silencing of *COMT1* in tomato decreased its melatonin concentration, by contrast, overexpressing this gene conferred heat stress tolerance along with an increase of melatonin accumulation^27,28^. Application of melatonin in agriculture is a new frontier, worthy of being explored. However, the knowledge of melatonin biosynthesis and physiological roles in plants was limited. A clear understanding of the functions of melatonin is necessary to decipher the metabolic network in C4 species, which have far-reaching values on genetic improvement of bioenergy crops. Here, we introduce the foreign genes *AANAT* and *HIOMT* derived from ovine into switchgrass to study the functions of melatonin on plant growth, development and stress tolerance.

## Results

### Transformation and molecular identification of transgenic switchgrass

**Figure 1:**
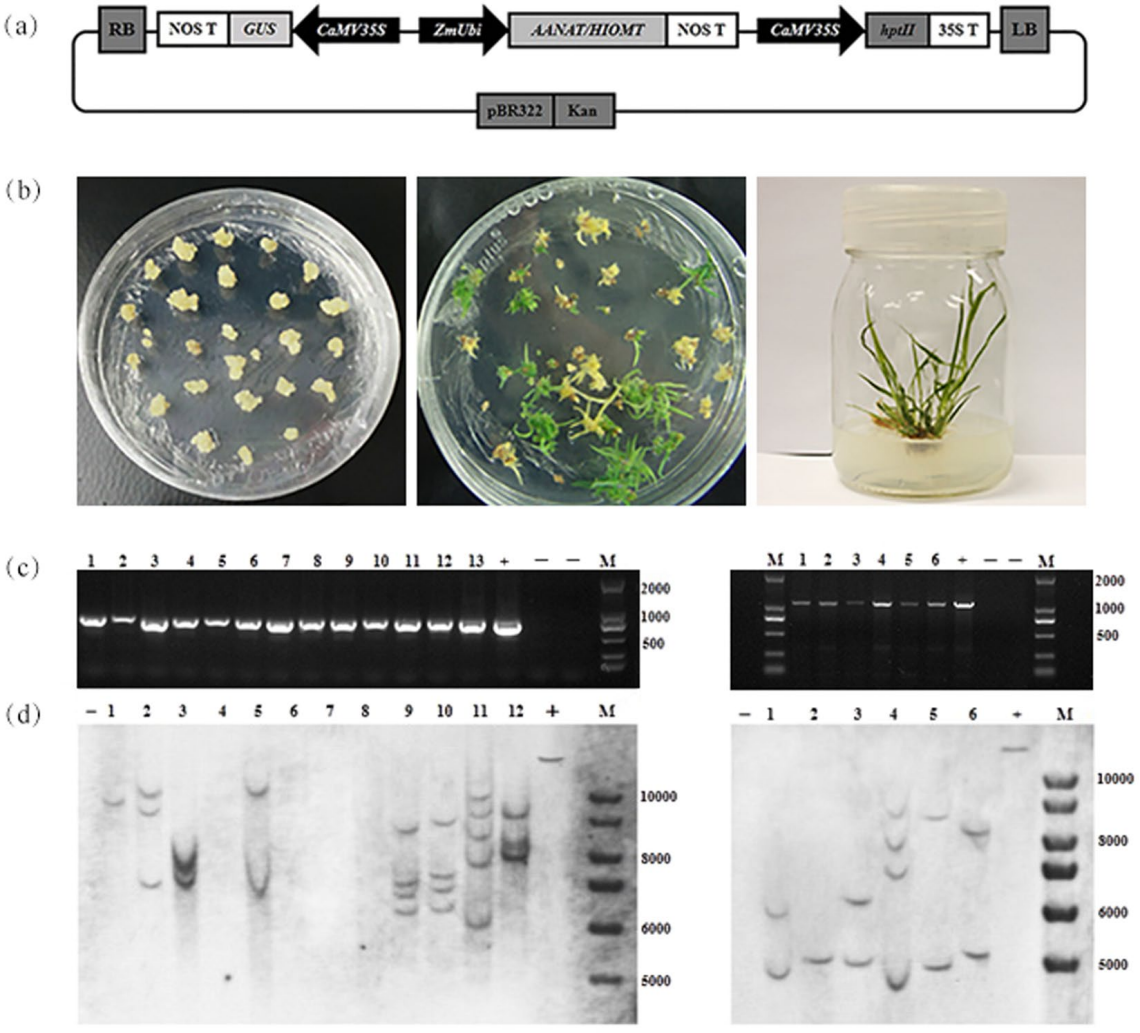
Stages of transformation and molecular identification of transgenic switchgrass. (**a**) Diagram depicting the overexpression vectors. (**b**) Stages in the process of switchgrass transformation. (**c**) The cropped gels of PCR analysis in transgenic plants. M: DL 2000 molecular weight marker; + : plasmid (positive control); -: wild type and H_2_O; lanes numbered 1–13 (left panel): transgenic plants expressing *oAANAT*; lanes numbered 1–6 (right panel): transgenic plants expressing *oHIOMT*. (**d**) The cropped blots of southern blot analysis. M: 1 kb plus DNA ladder; +plasmid (positive control); -wild type (negative control).

Transgenic plants were generated by transforming the binary vectors Ubi1301-*oAANAT* and Ubi1301-*oHIOMT* into lowland switchgrass cultivar Alamo. The transgenes were constitutively expressed under the control of maize ubiquitin promoter and 35 S promoter (Fig. 1a). Transgenic switchgrass were regenerated from embryogenic calli, which induced from switchgrass mature seeds and the resistant calli were transferred to regeneration medium for shoot induction. Roots and healthy plantlets were regenerated by transferring the shoots onto MS medium supplemented with the antibiotic (Fig. 1b).

The integration of the transgenes were confirmed in the resistant seedlings by polymerase chain reaction (PCR) and southern blot analyses. The putative transgenic plants were first examined by PCR using the primers designed from Ubi1301 vector. As shown in Fig. 1c, the expected sized fragments of Ubi1301-*oAANAT* (about 700 bp) and Ubi1301-*oHIOMT* (about 1100 bp) were amplified. For further confirmation, PCR positive plants were selected for southern blot analyses (Fig. 1d). One to five copies of *oAANAT* and *oHIOMT* genes were stably integrated into these transgenic lines. The full-length gels and blots were shown in the Supplementary Fig. S6. Results from both the PCR and Southern blot analysis confirmed that the *oAANAT* and *oHIOMT* genes were incorporated into the switchgrass genome.

### Effect of overexpressing *oAANAT* and *oHIOMT* on switchgrass growth and flowering

**Figure 2:**
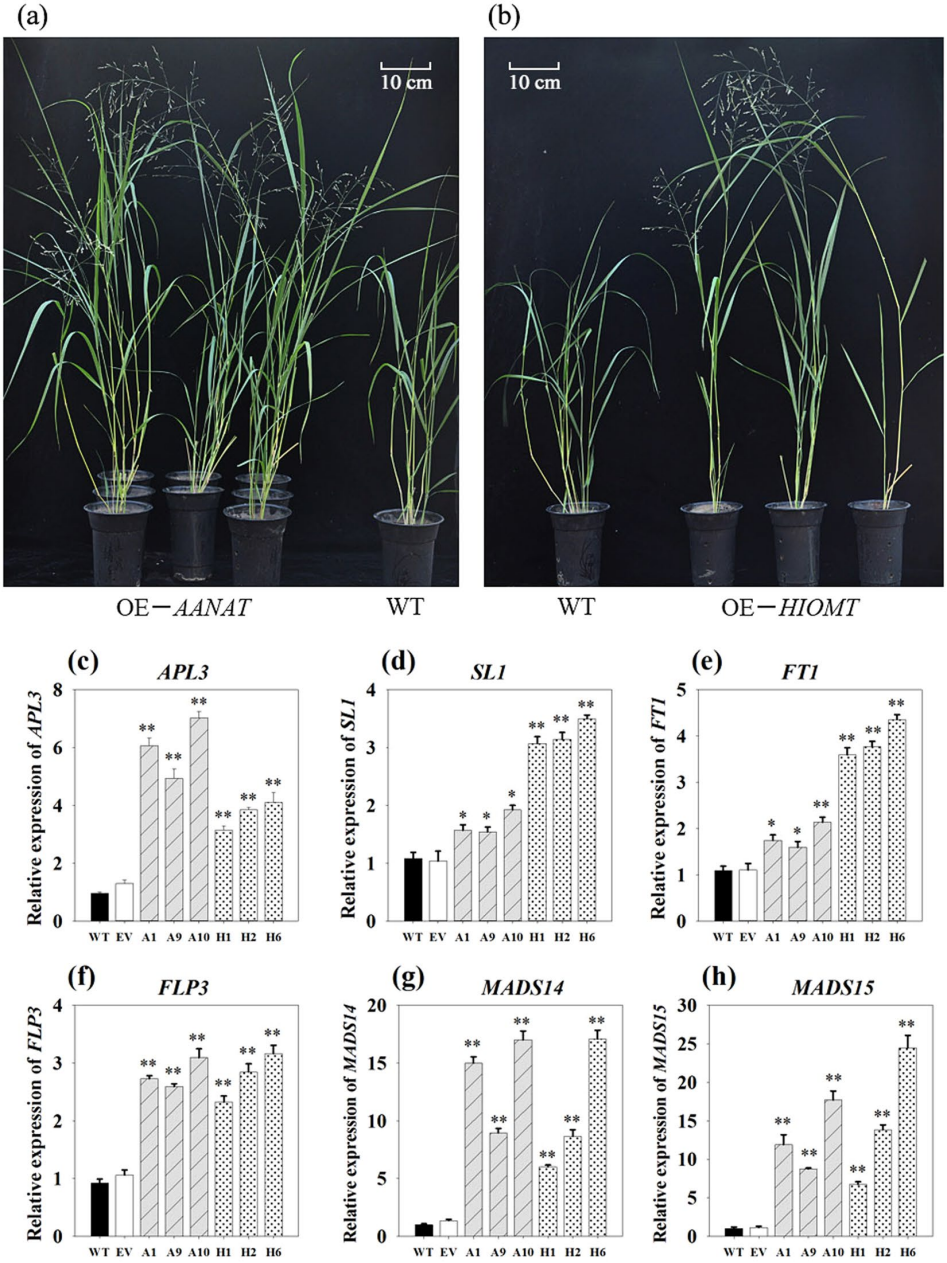
The phenotype comparision and expression levels analysis of flowering-related genes in transgenic switchgrass. (**a**,**b**): Phenotypes of switchgrass overexpressing *oAANAT* and *oHIOMT*. Expression levels of *APL1* (**c**), *SL1* (**d**), *FT1* (**e**), *FLP3* (**f**), *MADS14* (**g**), and *MADS14* (**h**). WT: wild type; EV: lines expressing the empty vector only; OE-*oAANAT*: transgenic plants of *oAANAT*; OE-*oHIOMT*: transgenic plants of *oHIOMT*; The data show the mean ± S.E. of triplicate measurements. * and ** indicate a significant difference from that of WT at *P < *0.05 and *P* < 0.01, respectively.

Transgenic lines as well as the control plants were subjected to phenotypic analysis. Interestingly, under the same greenhouse conditions, the transgenic plants showed obvious advantages with respect to plant height and flowering compared to the WT (Fig. 2a,b). The vertical height of WT and EV averaged 61.35 cm, whereas that of OE-*oAANAT* and OE-*oHIOMT* transgenic plants showed 1.10 to 1.64-fold and 1.12 to 1.31-fold increase compared to the two controls, respectively (Supplementary Fig. S1).

Morever, we found that genes involved in flowering, FLOWERING LOCUS T1 (*FT1*), APETALA 3 (*AP3*)-like gene (*APL3*), SUPPRESSION OF OVEREXPORESSION OF CONSTANS1 (SOC1)-like gene (*SL1*), FLOWERING PROMOTING FACTOR 3-like gene (*FLP3*) and MADS-Box gene (*MADS15*, *MADS6*) were significantly up-regulated (*P* < 0.05) in transgenic lines (Fig. 2c–h).

### Effect of overexpressing *oAANAT* and *oHIOMT* on melatonin content

**Figure 3:**
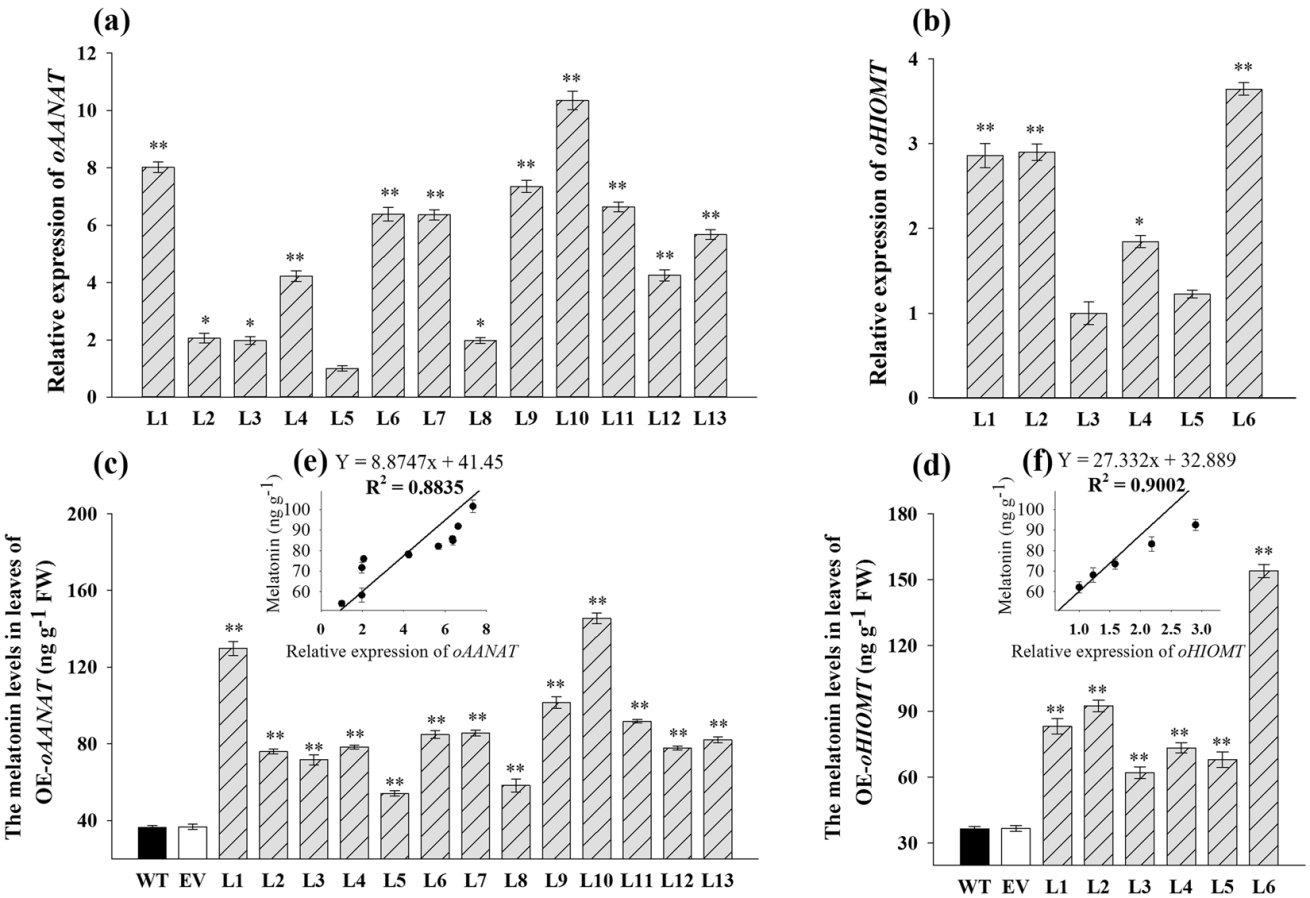
Detection of relative mRNA expression and melatonin levels in transgenic switchgrass leaves and their correlation analysis. (**a**,**b**) Relative expression of *oAANAT* (normalized to line5) and *oHIOMT* (normalized to line3) was detected by qRT-PCR; (**c**,**d**) Melatonin levels in different OE-*oAANAT* and OE-*oHIOMT* lines; (**e**,**f**) Correlation analysis of melatonin levels and relative expression of *oAANAT and oHIOMT*, respectively. The data represents the mean ± S.E. of triplicate experiments. * and ** indicate a significant difference from that of WT at *P* < 0.05 and *P* < 0.01, respectively.

The relative mRNA expression levels were detected by quantitative real-time PCR (qRT-PCR). In OE-*oAANAT* lines, line 10 exhibited the highest expression level, followed by lines 1 and 9 (Fig. 3a). Among the OE-*oHIOMT* transgenic lines, line 6 had the highest expression, followed by lines 2 and 4 (Fig. 3b). To determine whether the ectopic expression of *oAANA*T and *oHIOMT* genes affected the melatonin contents in switchgrass, the accumulation of melatonin was calculated. As shown in Fig. 3c,d, the melatonin content was significantly higher in the transgenic plants than in the control plants (*P* < 0.01). Melatonin content in the control seedlings averaged 36.58 ng g^−1^ fresh weight (FW), whereas the corresponding levels in OE-*oAANAT* lines varied from 54.15 to 145.42 ng g^−1^ FW. The increase in melatonin content in OE-*oAANAT* transgenic plants was in the range of 32–75% (Fig. 3c). Among the OE-*oHIOMT* transgenic lines, line 6 had an approximately four-fold increase in the melatonin content (153.98 ng g^−1^ FW) compared with the average content in control plants. The melatonin content in OE-*oHIOMT* transgenic lines increased by 42–76% (Fig. 3d).

To examine whether the melatonin content was associated with plant heights and gene expression, correlation analysis were performed. As shown in Supplementary Fig. S1c,d, plant heights were highly correlated with the melatonin levels (r^2^ = 0.80 and 0.94, respectively), except in the OE-*oHIOMT* line 6, which showed a decreased plant height compared to the control plants; this line was, therefore, not included in the correlation analysis. Also, the melatonin contents were highly correlated with the expression levels of *oAANAT* and *oHIOMT* genes (r^2^ = 0.88 and 0.90, respectively) (Fig. 3e,f). Thus, we confirmed that overexpression of *oAANAT* and *oHIOMT* genes in switchgrass resulted in the increase of endogenous melatonin content.

### Characterization of tissue-specific and spatial accumulation of melatonin

**Figure 4:**
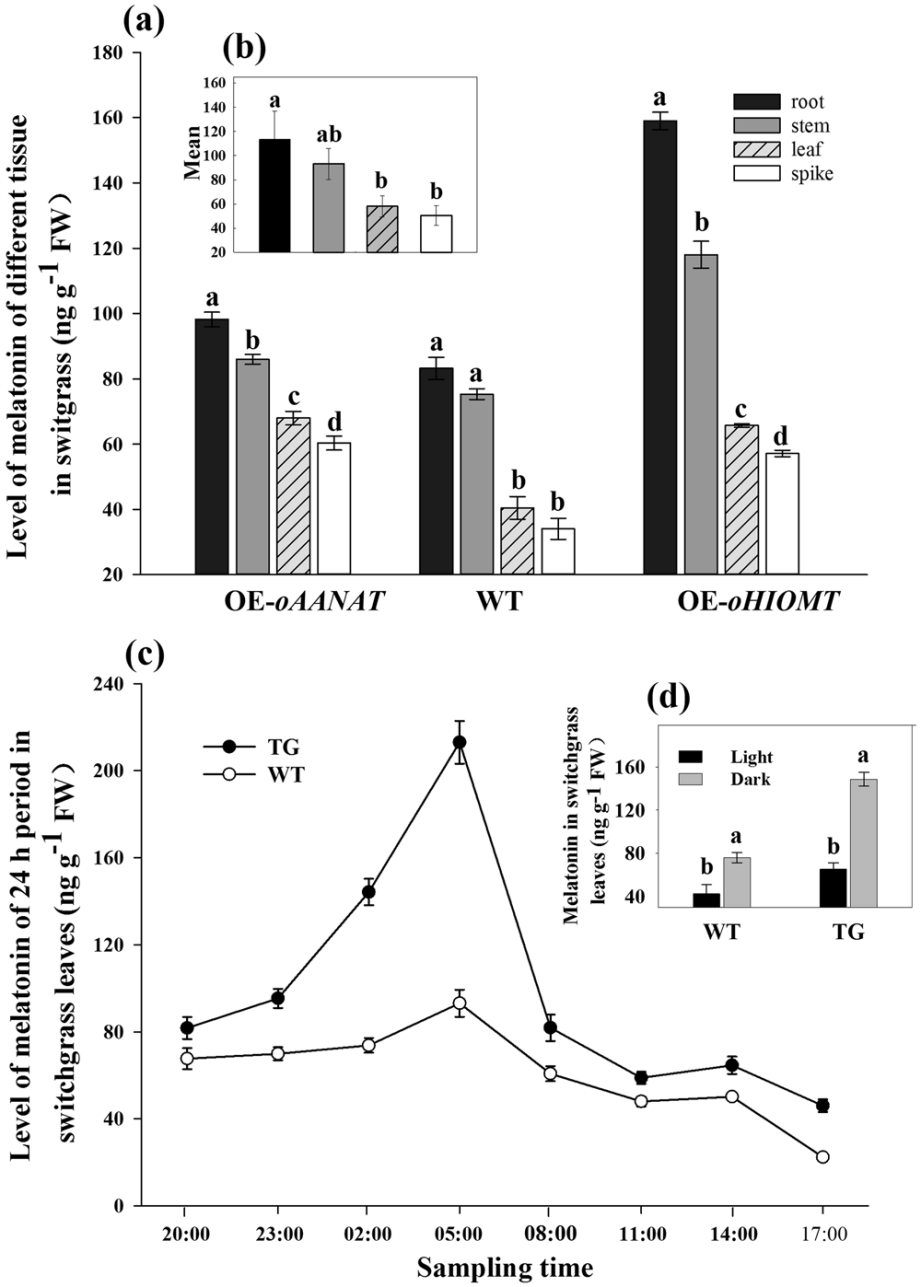
Detection of melatonin levels in different tissues and in different time points during the 24-h period. (**a**) Melatonin levels in root, stem, leaf, and spike. OE-*oAANAT*: transgenic plants of *oAANAT* (Line 13); (**b**) Average melatonin content in different tissues; (**c**) Melatonin levels of switchgrass leaves during the 24-h period; (**d**) Melatonin levels of switchgrass leaves in dark and light period. WT: wild type; OE-*oHIOMT*: transgenic plants of *oHIOMT* (Line 4); TG: transgenic plants of *oAANAT* (Line 1). The data show the mean ± S.E. of triplicate experiments. Columns with different letters indicate significant differences at *P* < 0.05.

To study the tissue-specific expression patterns, melatonin was isolated from different tissues (root, stem, leaf, and spike) of switchgrass, and quantified by HPLC. We found that melatonin accumulated to high levels in root and stem compared with other tissues in WT as well as in the transgenic plants (Fig. 4b). Compared with WT plants, the OE-*oAANAT* plants had melatonin levels that increased by 15%, 12%, 41%, and 44% in the root, stem, leaf, and spike, respectively, whereas the increase in the corresponding tissues of OE-*oHIOMT* plants were 48%, 36%, 38%, and 40%, respectively (Fig. 4a).

To reveal the circadian rhythm of melatonin in switchgrass, leaves of the transgenic lines (OE-*oAANAT*-line 1) and WT were assayed over a 24-h period. During this period, the accumulation patterns of melatonin at different time points were similar in WT and transgenic plants, with two peaks obtained at 05: 00 and 14: 00. However, at all the time points, the melatonin levels in the transgenic plants were higher than that in WT plants; especially during the period from 23: 00 to 05: 00, the melatonin levels dramatically increased in the transgenic plants, with the highest level appearing at 0500; the increase being 56% more than the increase observed in WT (Fig. 4c). Moreover, we also compared the melatonin level at various time points during the dark (20: 00, 23: 00, 02: 00, and 05: 00) and light (08: 00, 11: 00, 14: 00, and 17: 00) periods. As shown in Fig. 4d, samples harvested during the dark period contained more melatonin content than those harvested during the day in both the WT and transgenic plants. In OE-*oAANAT*-line 1, the melatonin level under dark phase (148.57 ng g^−1^ FW) showed 2.27-folds higher level compared to that under light phase (65.34 ng g^−1^ FW), whereas in WT, the corresponding level was only 1.79-fold higher.

### Effects of salinity on plant phenotype and growth

**Figure 5:**
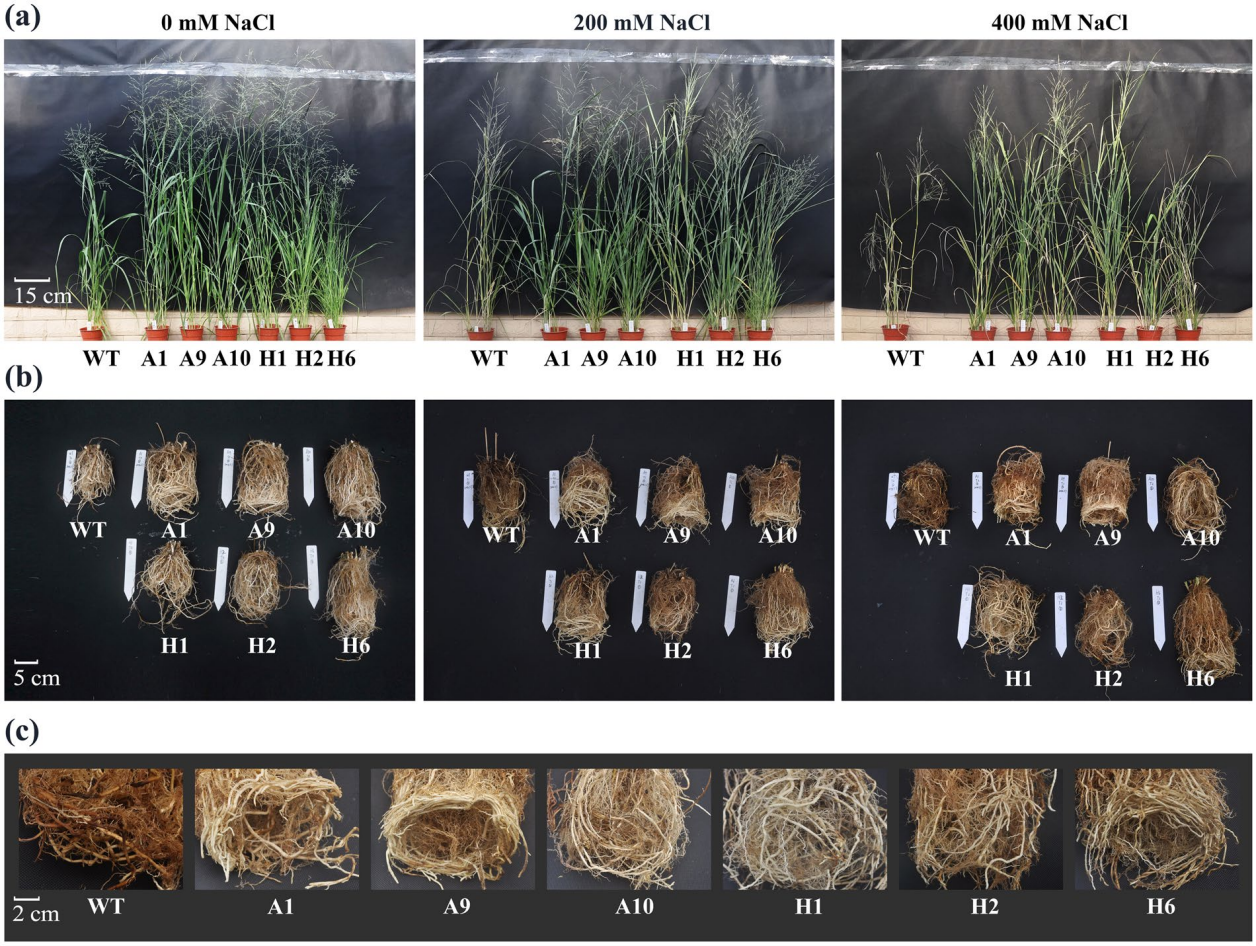
Phenotypic analyses of different transgenic lines under NaCl stress for 40 days. The changes in phenotypic characters of shoot (**a**) and root (**b**) under different salt stress conditions; (**c**) The details of root changes involved in 400 mM NaCl conditions; Plants were treated with 0, 200 and 400 mM NaCl in greenhouse. WT: wild type; A1, A9 and A10: the transgenic plants overexpressing o*AANAT*; H1, H2 and H6: the transgenic plants overexpressing o*HIOMT*.

We analyzed the phenotype and growth performance of TG and WT plants under various concentrations of NaCl (0, 200 and 400 mM) for 40 days. After grown in normal (i.e. unstressed; 0 mM NaCl) condition, both transgenic and control plants grew vigorously and no visible phenotypic differences were observed. Under 400 mM NaCl treatment, the leaves of WT plants, particularly the older leaves showed severe withering (Fig. 5a), and the roots were almost died (Fig. 5b), whereas these symptoms were effectively alleviated in transgenic lines (Fig. 5).

Additionally, based on the relative increase of growth parameters with and without salt stress treatments, transgenic plants showed obvious advantages on growth under salt stress (Table 1). Among the transgenic lines, H6 produced more tillers, A1 and A10 showed obvious advantages on plant height and stem diameter, H1 and H2 exhibited better growth performance on leaf width. Furthermore, dry weights (DW) of all plants decreased with increasing concentration of NaCl, but that of transgenic lines was higher than control plants. These results confirmed that overexpression of *oAANAT* and *oHOMT* triggered a significant increase in switchgrass growth and biomass under salt treatment.

**Table 1:** Effects of salinity on the growth performance and dry biomass yield of wild-type and transgenic switchgrass.

	Tiller number	Plant height (cm)	Leaf width (cm)	Stem diameter (cm)	Dry Weight (g)	
					Shoot	Root
**0 mM NaCl**						
WT	7.7 ± 0.3 c	14.67 ± 0.58 c	1.05 ± 0.03 c	0.53 ± 0.01 c	26.15 ± 0.15 d	11.81 ± 0.42 d
A1	10.7 ± 0.7 bc	28.83 ± 0.73 a	1.81 ± 0.06 b	0.80 ± 0.04 a	36.72 ± 0.41 a	16.68 ± 0.17 a
A9	9.1 ± 0.6 c	22.73 ± 0.43 b	1.63 ± 0.03 b	0.62 ± 0.01 b	28.46 ± 0.24 c	14.62 ± 0.28 b
A10	12.3 ± 0.6 b	27.31 ± 0.82 a	1.67 ± 0.03 b	0.88 ± 0.03 a	36.86 ± 0.44 a	16.65 ± 0.17 a
H1	8.3 ± 0.3 c	23.33 ± 0.68 b	2.07 ± 0.07 a	0.66 ± 0.02 b	29.82 ± 0.37 b	14.61 ± 0.16 b
H2	8.1 ± 0.6 c	23.97 ± 0.49 b	2.17 ± 0.12 a	0.73 ± 0.03 a	29.20 ± 0.38 bc	12.78 ± 0.12 c
H6	22.3 ± 0.7 a	15.47 ± 0.67 c	1.17 ± 0.07 c	0.54 ± 0.02 c	26.29 ± 0.51 d	14.86 ± 0.14 b
**400 mM NaCl**						
WT	2.2 ± 0.1 c	3.43 ± 0.34 f	0.23 ± 0.03 d	0.11 ± 0.02 e	13.34 ± 0.22 f	6.24 ± 0.19 c
A1	5.3 ± 0.3 b	13.03 ± 0.35 a	0.73 ± 0.03 ab	0.34 ± 0.02 c	22.11 ± 0.14 b	12.21 ± 0.12 a
A9	5.1 ± 0.6 b	9.57 ± 0.52 c	0.67 ± 0.09 ab	0.33 ± 0.01 c	20.42 ± 0.15 c	12.44 ± 0.04 a
A10	5.7 ± 0.3 b	11.62 ± 0.26 b	0.63 ± 0.03 b	0.54 ± 0.03 a	24.57 ± 0.14 a	10.67 ± 0.09 b
H1	5.2 ± 0.1 b	9.37 ± 0.19 c	0.83 ± 0.03 ab	0.42 ± 0.01 b	21.74 ± 0.13 b	12.05 ± 0.11 a
H2	5.3 ± 0.1 b	8.01 ± 0.38 d	0.87 ± 0.12 a	0.56 ± 0.01 a	19.61 ± 0.21 d	10.28 ± 0.04 b
H6	15.7 ± 0.3 a	5.63 ± 0.23 e	0.43 ± 0.03 c	0.23 ± 0.01 d	17.69 ± 0.16 e	11.91 ± 0.06 a

The data show the mean ± S.E of three replicate samples. The letters following the value in the same row indicate the differences of plants under same condition according to ANOVA analysis (*P* < 0.05). The relative increase in growth parameters (Tiller number, Plant height, Leaf width, Stem diameter) were calculated using the formula: increased growth parameters = TG/WT_40_ − TG/WT_0_. (TG/WT_40_ represents data were measured after 40 days of salt treatment, TG/WT_0_ represents data were measured just before salt treatment).The dry weight (DW) of shoot and root were measured after 30 days of salt-treatment.

### Effects of salinity on plant physiological traits

**Figure 6:**
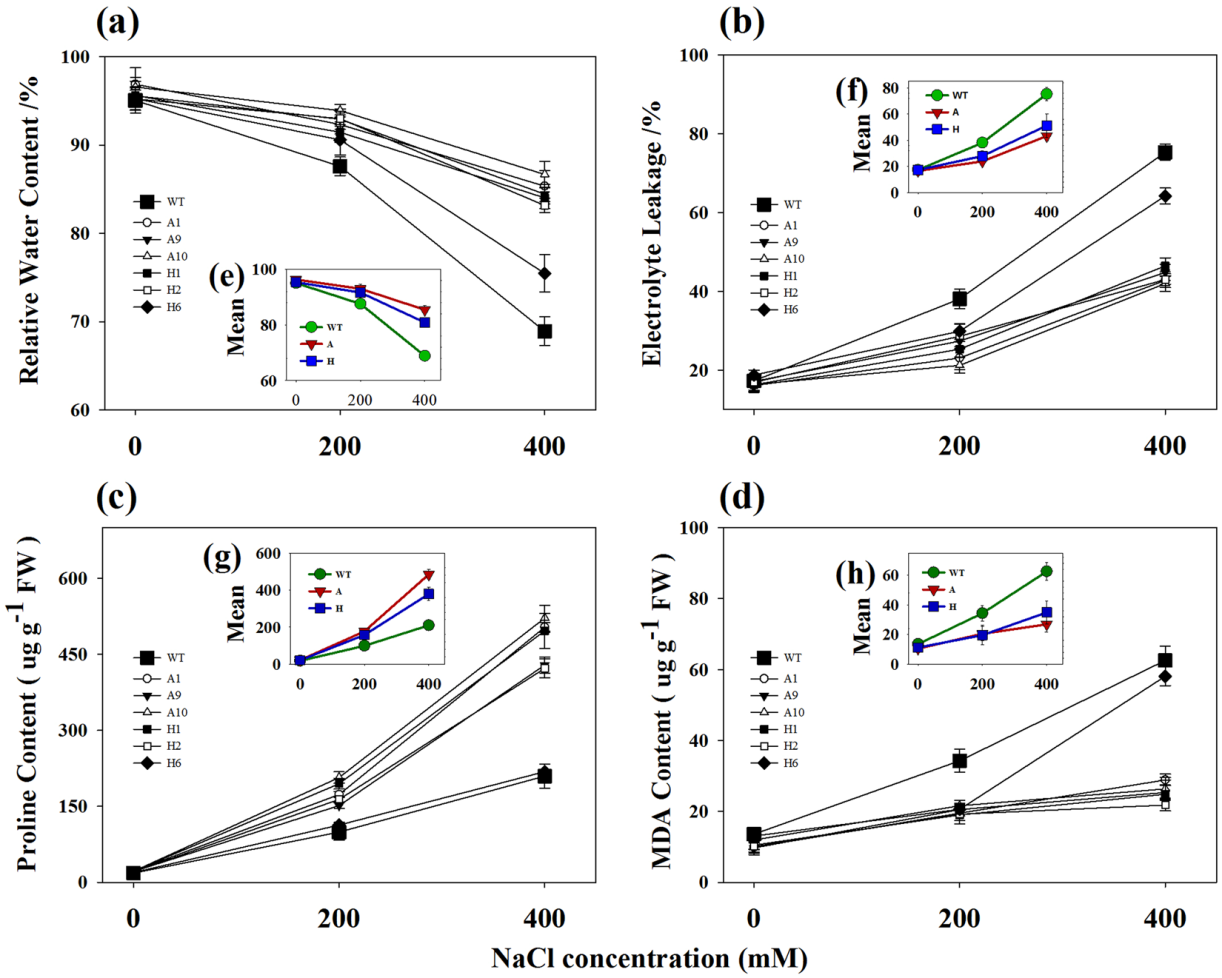
Physiological analyses of WT and transgenic switchgrass under different concentrations of salt stress for 40 days. The changes of RWC (**a**), EL (**b**), proline (**c**) and MDA content (**d**) under salt stress conditions; Average content of RWC (**e**), EL (**f**), proline (**g**) and MDA (**h**); The data show the mean ± S.E of three replicate samples.

We performed physiological assessment of transgenic lines (A1, A9, A10, H1, H2, H6) and WT plants under various salt stress conditions (0, 200, 400 mM NaCl). Transgenic lines and WT plants exhibited approximately physiological status regarding RWC, EL, MDA and proline contents under normal physiological conditions. However, under salt stress, considerable differences were observed between transgenic lines and WT plants (Fig. 6). Transgenic switchgrass lines, H6, which exhibited the highest level of melatonin but the lowest tolerant to salt stress; not included in the following analysis.

Considerable reduction of RWC was observed when WT plants were grown under 200 or 400 mM NaCl for 40 days, whereas the decrease in TG plants averaged only 3% and 11%, respectively (Fig. 6a,e). The EL of the WT plants increased to 38% and 75% under 200 mM and 400 mM NaCl conditions, respectively, while that of TG plants was only 25% and 43%, respectively (Fig. 6b,f). As compared with the WT plants, the content of proline increased by 2.40, 2.04, 2.49, 2.37 and 2.01-fold in transgenic lines A1, A9, A10, H1, and H2 under 400 mM NaCl conditions, respectively (Fig. 6c,g). Salinity substantially increased MDA content in WT plants. As shown in Fig. 6d,h, the increase of MDA in WT plants was 3.58 and 4.16-fold compared to TG plants under 200 mM and 400 mM NaCl conditions, respectively.

Moreover, the RWC, EL, MDA, and proline contents of transgenic lines and WT plants were also compared during salt stress for 0, 10, 20, 30 and 40 days. As shown in Supplementary Fig. S2, no considerable difference was observed when plants exposed to 400 mM NaCl for 0 days. However, with prolongation of treatment time, substantially higher RWC and proline content were observed in transgenic lines. Meanwhile, transgenic lines showed less cell membrane damage and lower lipid oxidative level compared to WT plants (Supplementary Fig. S2).

We also measured leaf chlorophyll content of both transgenic lines and WT plants under different salt stress conditions. The total chlorophyll content in the OE-o*AANAT* averaged 2.91, 3.23, and 2.44 mg g^−1^ FW under 0, 200, and 400 mM NaCl conditions, respectively, whereas the corresponding lever was 2.06, 1.71, and 1.01 mg g^−1^ FW in the WT plants (Supplementary Fig. S3). The transgenic lines (H1, H2, H6), overexpressing o*HIOMT*, showed a similar leaf chlorophyll as those OE-o*AANAT*, and exhibited 1.33, 1.81 and 2.45-fold higher level compared with WT plants under 0, 200, and 400 mM NaCl conditions, respectively (Supplementary Fig. S3). It was noticed that a lower level of salt stress (200 mM NaCl) induced an increase of chlorophyll content in transgenic lines.

### Effects of salinity on plant Na^+^ and K^+^ accumulation

**Figure 7:**
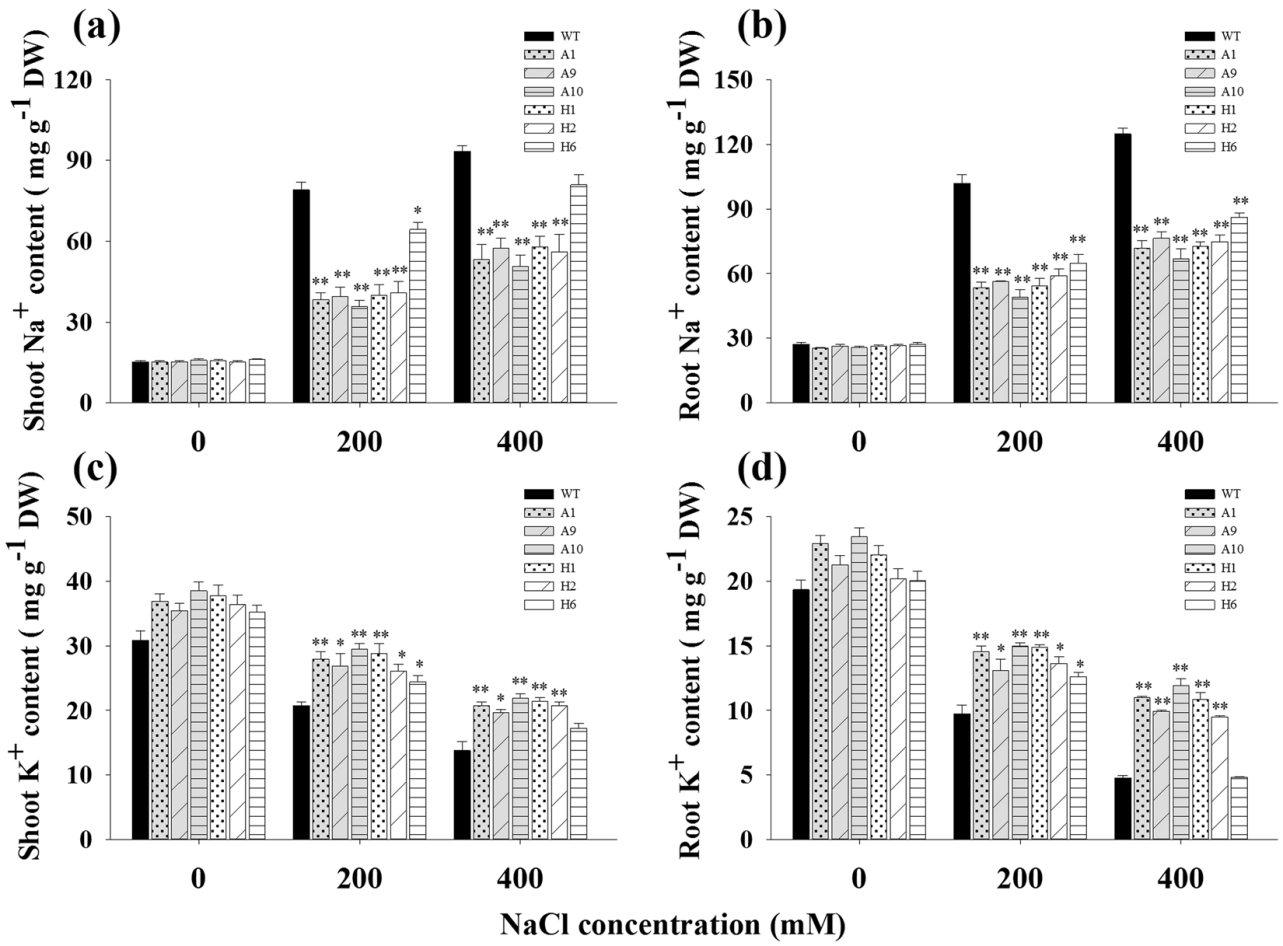
Na^+^ and K^+^ contents in tissues from WT and transgenic switchgrass under different concentrations of salt stress. (**a**) Na^+^ content in shoots; (**b**) Na^+^ content in roots; (**c**) K^+^ content in shoots; (**d**) K^+^ content in roots; The data show the mean ± S.E of three replicate samples. *Indicates significant differences from the WT at *P* < 0.05; **Indicates significant differences from the WT at *P* < 0.01.

Total Na^+^ and K^+^ analysis was performed using the shoots and roots of WT and TG plants after salt stress treatment. As shown in Fig. 7, The TG and WT plants exhibited approximately equal Na^+^ and K^+^ contents under normal condition. Under salt treatment, the Na^+^ concentrations increased in the shoots and roots of all plants, but the increases in WT plants were considerably higher than that in transgenic lines. For instance, under 200 mM NaCl treatment, WT plants accumulated 2.09- and 1.87-fold higher Na^+^ in shoots and roots respectively, compared to transgenic lines (Fig. 7a,b). In contrast, a decrease of K^+^ contents in shoots and roots of all plants was observed under salt treatment, whereas the decrease was more substantially in WT plants. As shown in Fig. 7c,d, under 400 mM NaCl condition, transgenic lines showed considerably higher K^+^ content compared to WT plants and exhibited 1.51- and 2.22-fold in shoots and roots, respectively.

The Na^+^ contents accumulated to high levels, while the K^+^ contents decreased in WT as well as in the transgenic plants, which resulted in a decreased of K^+^/Na^+^ ratios under salt stress (Supplementary Fig. S4). However, the K^+^/Na^+^ ratio of shoots and roots in transgenic lines were higher than that in WT plants, especially under 200 mM NaCl treatment. The ratio in shoots and roots in the TG plant averaged 0.73 and 0.26, respectively, whereas that in the corresponding tissues of WT plants were only 0.26 and 0.09, respectively (Supplementary Fig. S4). The K^+^/Na^+^ ratio in shoots was considerably higher than in roots. These results indicated that overexpression of o*AANAT* and *oHIOMT* genes in switchgrass is beneficial for plants to maintain a higher K^+^/Na^+^ ratio in both shoots and roots.

### Effects of salinity on gene expression

In this report, we observed that the transgenic switchgrass accumulated more K^+^ and less Na^+^ compared to WT plants under salt stress, indicating that the enhanced salt tolerance in transgenic switchgrass may associated with the Na^+^ exclusion or vacuolar compartmentation mechanism. Vacuolar Na^+^/H^+^ antiporter gene (*PvNHX1*), which contributed to compartmentalize excess Na^+^ into vacuoles, was also assayed in this study. As shown in Supplementary Fig. S5, the expression level of *PvNHX1* was induced at 200 mM NaCl treatment and obviously decreased at 400 mM NaCl in both TG and WT plants. There were obvious differences in the expression levels of *PvNHX1* in these transgenic lines. *PvNHX1* transcript was significantly higher in the transgenic plants (A10 and H1) than that in the non-transgenic plants, and it also displayed a highly correlation with the expression of *oAANAT* and *oHIOMT* among all the lines, except that in the H6, which showed the highest expression of *oHIOMT* but lowest expression of *PvNHX1*; therefore, the salt tolerance of H6 line was not improved.

## Discussion

Genetic manipulations to elevate the melatonin production by introduction of the genes from other species, even with low homology have proven to be feasible^8,13,16,17^. In the present study, we introduced *AANAT* and *HIOMT* genes from the pineal gland of *Ovis aries* into switchgrass. Similar to the observation in other studies, melatonin content was significantly higher in the transgenic plants than that in the non-transgenic plants (*P* < 0.01) (Fig. 3c,d). The accumulation of melatonin was highly correlated with the expression level of transgenes in different lines (Fig. 3e,f), indicating that the increase of melatonin levels in transgenic switchgrass plants directly resulted from the ectopic expression of *oAANAT* and *oHIOMT* genes. This result is in agreement with that reported by Park *et al*.^29^. It also indicated that the ectopic expression of *oAANAT* and *oHIOMT* have an analogous effect on plant melatonin synthesis regardless of the host species and suggested that there might be a similar regulatory mechanism for melatonin biosynthesis in animals and plants.

Melatonin has been found in different plant species and its levels vary greatly. Generally, medicinal herbs and coffee seeds contain the highest level of melatonin, which sometimes accumulates up to 3800 and 5800 ng g^−1^ DW, respectively^30,31^. However, in most plant species, including rice, sweet corn, lupin, and tomato, melatonin concentrations reached only a few nanograms per gram fresh mass^32^. In the present study, the melatonin of WT and EV totaled 36.46 and 36.69 ng g^−1^ FW, respectively (Fig. 3c,d), which was much higher than that in the other common crops. This might explain the wide adaptation of switchgrass to marginal soils. Besides, melatonin has also been detected in almost all the tissues in switchgrass (Fig. 4a) and a relatively high level was detected in the root and stem compared with leaf and spike in both the transgenic lines and WT plants. Similar patterns have been detected in tomato (*Solanum lycopersicum* L.)^32^ and morning glory (*Pharbitis nil* Choisy ‘Violet’)^33^. Because the leaves and spikes are directly exposed to the sunlight, whereas the roots and stems are always in darkness, it would be expected that melatonin synthesis is involved in dark signaling. This speculation was supported by the findings in St John’s wort (*Hypericum perforatum* L.), in which the etiolated hypocotyls germinated in completely dark condition showed a 15-30-fold increase in melatonin concentration over any light-adapted tissue^34^. In the present study, the possible role of melatonin as a regulator of light/dark cycles was studied. We observed two peaks of melatonin (at 05: 00 and 14: 00, respectively) during a 24-h period in switchgrass (Fig. 4c). This result was consistent with previous reports^16,23,35^, who believed that the “double peak” phenomenon was attributed to both endogenous and environmental factors^16^. Our results suggested that the first peak could be the result of the plant’s biological clock and was induced by darkness. Similarly, in grape fruit, the melatonin peak occurred at dawn, as well^36^. The second peak might have been the result of high temperature and UV radiation, because during the day, the highest temperature and maximum light intensity are present at around 14: 00. Previous studies where the melatonin synthesis was induced by high temperature and UV radiation to protect the plants against the damage of ROS support this conclusion^36^. Moreover, our results showed that the melatonin content was considerably higher in dark periods than in the daylight (Fig. 4d), suggesting that the melatonin in switchgrass has the potential for diurnal or circadian rhythms as demonstrated in other species^6,16,35,37^. Perhaps, the relation between melatonin and photosynthesis factors will break through the original study barriers and open a new field of research.

It is intriguing to explore the phenotype of the transgenic switchgrass overexpressing the two ovine genes. In this study, the growth of transgenic plants in both OE-*oAANAT* and OE-*oHIOMT* lines was stimulated relative to the WT and EV plants (Fig. 2). The growth rate was highly correlated with melatonin accumulation in the different lines (Supplementary Fig. S1). However, this was excepted for the OE-*oHIOMT* line 6, which exhibited the highest level of melatonin but the lowest plant height, indicating that melatonin played different roles in plant growth and developmental regulation at low and high concentrations. This speculation was supported by the findings in lupin (*Lupinus albus* L.), in which melatonin induced active growth of hypocotyls at micromolar concentrations, whereas it had a growth-inhibitory effect at high concentrations^38^. Similar results were observed in mustard (*Brassica juncea*) roots^39^. Additionally, melatonin has been proposed to play a significant role in the regulation of reproductive physiology and flower development^22,40,41^. Some results have proven that melatonin delayed flowering and lowered the percentage of flowering plants in some early steps of the transition to flowering. In contrast to these conclusions, we observed the transgenic lines flowered early than WT (Fig. 2). Transcript levels analysis in our study confirmed and strengthened this notion. The key flowering-time regulators, such as *FT1*, *APL3*, *SL1*, *FLP3*, *MADS6* and *MADS15* were significantly up-regulated (P < 0.05) in transgenic switchgrass (Fig. 2c–h). It should be emphasized that previous studies in flowering mainly focused on exogenous melatonin application, and the concentrations required for the inhibition of flowering exceeded the naturally occurring levels by several orders of magnitude. It is likely that the metabolic pathways of the endogenous and exogenous melatonin were not completely consistent. Moreover, previous reports regarding melatonin on flowering were focus on C3 species, such as rice^22^. C4 plants have lots of different physiological functions. In grass, the effect of melatonin has been reported in bermudagrass through exogenous application^42^. However, this is the first time we provide the direct evidence for functional study of melatonin on C4 grass species by genetic modification. Further studies are still needed to identify the exact mechanism of melatonin action on growth and flowering.

Melatonin, a potent free-radical scavenger, is also believed to function as the first line of defense against internal and environmental stress. Several studies have confirmed that melatonin can protect plants against damage caused by adverse environmental conditions^13,16,18,19^. Consistent with those earlier conclusions, we found that transgenic switchgrass showed better phenotype (Fig. 5), growth performance (Table 1) and maintained an improved physiological capacity (Fig. 6, Supplementary Fig. S2) under high-salinity conditions. Moreover, transgenic switchgrass accumulated higher K^+^ and lower Na^+^ levels in all tissues compared with WT plants (Fig. 7, Supplementary Fig. S4), indicating that transgenic plants have developed specific mechanisms, such as Na^+^ exclusion or vacuolar compartmentation. It would be expected that the enhanced salt tolerance in transgenic switchgrass may associated with the vacuolar Na^+^/H^+^ antiporter gene (*PvNHX1*). This speculation was supported by the results that the relative expression of *PvNHX1* was greatly up-regulated and maintained considerably higher levels in transgenic lines during salt treatment (Supplementary Fig. S5). This result is in agreement with that reported by Li *et al*.^43^. Additionally, the transgenic switchgrass with high levels of melatonin showed elevated chlorophyll synthesis during salt stress, suggesting a role for melatonin in delaying leaf senescence and maintaining high photosynthetic efficiency (Supplementary Fig. S3). Similar results were observed in transgenic rice plants under cold stress^25^ and green macroalga (*Ulvasp*.) exposed to toxic conditions^44^, barley leaves^21^ and apple leaves during senescence^45^.

In summary, we demonstrate that genetic manipulations to elevate melatonin production were feasible. Melatonin showed obvious tissue-specific expression patterns and circadian rhythms in switchgrass. Besides, transgenic switchgrass with high levels of melatonin exhibited promoted growth performance, earlier flowering and improved salt resistance compared with non-transgenic plants. Our data will provide a valuable foundation for further study of the potential role of melatonin in plants.

## Methods

### Transformation and molecular identification of transgenic switchgrass

The plasmids containing *oAANAT* and *oHIOMT* were provided by the Key Laboratory of Animal Genetics and Breeding of China Agricultural University (Beijing). The open reading frames (ORFs) of *oAANAT* and *oHIOMT* genes were amplified using specific primers *oAANAT*-F1, *oAANAT*-R1 and *oHIOMT*-F1, *oHIOMT*-R1, respectively, designed based on the sequences of *oAANAT* (GenBank accession no. NM_001009461.1) and *oHIOMT* (GenBank accession no. JF815374.1) (Supplementary Table S1). The binary expression vector Ubi1301 (provided by the Sinogene Scientific Company) was used for switchgrass transformation.

Transgenic switchgrass was regenerated from embryogenic calli derived from mature seeds as described by Liu *et al*.^46^. The genetic transformation was performed by the *Agrobacterium*-mediated method. After infection, co-cultivation, preculture and screening with hygromycin resistance, resistant calli were grown in 1/2 MS medium supplemented with 200 mg/L timentin and 50 mg/L hyg B for rooting. Finally, the rooted plantlets were transferred to a greenhouse for further growth. Wild-type (WT) plants regenerated from untransformed calli and transgenic plants with empty vector (EV) were both taken as controls.

Total genomic DNA was extracted from the transgenic (TG) and wild-type (WT) plants using the cetyltrimethyl ammonium bromide (CTAB) method^47^. To primarily screen the positive transgenic plants, a fragment of the expression vector Ubi1301 was amplified by using the specific primers Ubi1301-F and Ubi1301-R (Supplementary Table S1). To further examine the copy number of the integrated gene, Southern blot analysis was performed according to procedure described by Liu *et al*.^46^. Hygromycin phosphotransferase (*hyg*) gene obtained by PCR amplification of genomic DNA using specific primers *hyg*-F and *hyg-*R (Supplementary Table S1) was chosen as the probe. Probe labeling and hybridization were performed following the instructions provided in the DIG Labeling and Detection starter kit II (Roche Applied Science, Mannheim, Germany). The Ubi1301-*oAANAT* and Ubi1301-*oHIOMT* plasmids were used as the positive control.

### Gene expression and phenotypic analyses of transgenic switchgrass

Transgenic and WT plants were transplanted into plastic pots containing a compound medium of soil: vermiculite: humus [1:1:1 (v/v/v)] on the same day and grown under the same greenhouse environment (16/8 h light/dark cycle) in Beijing. When switchgrass grew to reproductive 3 (R3) stage (spikelets was fully emerged and peduncle could be seen)^48^, the transgenic and control lines (WT, EV) were subjected to phenotypic analysis. Plant height, tiller number, and flowering phenotypes were compared and calculated.

To determine the expression level of *oAANAT* and *oHIOMT* in the transgenic lines, qRT-PCR were conducted. Total RNA from the leaves was isolated using the TRIzol reagent method (Invitrogen, Carlsbad, CA, USA). First-strand cDNA was synthesized using PrimeScript^TM^ RT reagent kit with gDNA Eraser kit (TaKaRa, Shiga, Japan). qRT-PCR were performed using gene specific primers *oAANAT*-F2, *oAANAT*-R2 and *oHIOMT*-F2, *oHIOMT*-R2, respectively (Supplementary Table S1). The switchgrass *ubiquitin-1* (*PvUBQ1*) gene (GeneBank accession number: FL899020) was selected as the internal control and was amplified using primers *PvUBQ1*-F and *PvUBQ1*-R (Supplementary Table S1). The relative expression levels of *oAANAT* and *oHIOMT* genes were calculated using the 2^−ΔΔCt^ method^49^.

To determine the molecular mechanism underlying early flowering, three flowering-related genes (*FT1*, *APL1*, *SL1*) which have been functionally identified as key flowering regulators in switchgrass^50^, were chosen for gene expression analyses. Moreover, three potential flowering-related genes were also searched in switchgrass genomics resource (https://phytozome.jgi.doe.gov/pz/portal.html#!info?alias=Org Pvirgatum) using the rice (*Oryza sativa* L.) proteins as a BLAST query^51^. Internode 3 from control plants (WT and EV) and transgenic lines (A1, A9, A10, H1, H2 and H6) were collected when transgenic switchgrass grew to R3 stage. *PvUBQ1* was used for internal control. All primers used were listed in Supplementary Table S1.

### Determination of melatonin by HPLC

Melatonin was extracted and detected by High-Performance Liquid Chromatography (HPLC), according to a modification of the method described by Wang *et al*.^26^. The leaves of transgenic and control plants grown in a natural light cycle were collected at the same time, about 20:00. The recovery rate was determined by adding 150 µL 1 µg/mL melatonin standard (Sigma, St. Louis, MO, USA) into the suspension of samples before melatonin extraction using the methods described by Zhao *et al*.^23^. All measurements were performed in triplicate. The melatonin content was calculated by comparing the sample peak area (% fluorescence) with the standard curve of melatonin.

Besides determining the melatonin level in the selected transgenic switchgrass and WT plants, we also detected it in different tissues (root, stem, leaf, and spike) of OE-*oAANAT*-line 13 and OE-*oHIOMT*-line 4; these two lines had an intermediate level of melatonin among all the candidate transgenic plants. Leaves, stems, roots, and spikes were collected using the methods described above and immediately frozen at −80 °C until melatonin extraction.

For characterization of the circadian rhythm of melatonin in switchgrass, leaves of OE-*oAANAT* -line 1 and WT plants were assayed over a 24-h period to look for changes in the melatonin levels with respect to the light/dark cycle. Leaves were collected every three hours in the time course of 24 h (We started from 11: 00 on the first day and ended at 08: 00 on the second day). Two gram of fresh leaves were collected at each time point, were frozen in liquid nitrogen immediately, and stored at −80 °C until analysis.

### Plant materials and salt-stress treatments

At E5 stage, transgenic lines overexpressing *oAANAT* (A1, A9, A10), *oHIOMT* (H1, H2, H6) and WT plants were removed from soil, and consistent tillers were trimmed to sand culture for salt-stress treatments. They were watered with 1/2 × Hoagland nutrient solution supplemented with various concentrations of NaCl (0, 200 and 400 mM) and maintained for 40 days. The morphological differences were compared and recorded. Physiological indexes, such as relative water content (RWC), electrolyte leakage (EL), malondialdehyd content (MDA), proline content, total chlorophyll and ion content (Na^+^, K^+^) were analyzed. Gene expression (*PvNHX1*) under salt stress were investigated.

### Measurement of growth parameters

The transgenic lines overexpressing *oAANAT* and *oHIOMT* and the WT plants grown on 0 and 40 days after transplanting were used for determination of morphological difference. The relative increase in growth parameters (plant height, tiller number, leaf width, stem diameter) with and without salt were compared and calculated using the formula: TG/WT_40_-TG/WT_0_. Internode 3 (I3) was used for measuring stem diameter. The leaves of I3 were used to measure leaf blade width. Plant shoot and root were harvested separately for biomass detection.

### Physiological analysis of transgenic switchgrass

Mature leaves of transgenic lines and WT plants were collected every ten days for physiological analysis. RWC were measured according to method described by Bao *et al*.^52^. EL was measured to evaluate the stability of the plant cell membrane following the description of Lutts *et al*.^53^. MDA was determined using the thiobarbituric acid (TBA) reaction as described by Peever & Higgins^54^, and absorbance values were recorded at 532 nm, 450 nm and 600 nm. Proline was extracted with 3% sulfosalicylic acid and determined as described by Bates *et al*.^55^. After 40 days of treatment, the leaves of switchgrass were harvested for chlorophyll detection. Chlorophyll content was determined according to a modified method of Hartmut^56^. Furthermore, the shoot and root of WT and transgenic lines were harvested for Na^+^ and K^+^ detection respectively, using the method described by Wang & Zhao^57^.

### Analysis of gene expression under salt stress

Leaves of WT and transgenic lines were used for determination of gene expression in response to salt stress. *PvNHX1* (GeneBank accession number: KJ739865), we previously isolated from switchgrass, used specific primers *PvNHX1*-F and *PvNHX1-*R (Supplementary Table S1). The qRT-PCR was conducted as described above.

### Statistical analysis

All data are presented as means ± SE of triplicate samples and were analyzed through ANOVA using the SPSS software (Version 18.0, IBM, Armonk, NY, USA). A P-value < 0.05 or P < 0.01 was considered statistically significant. Fig.s were created using SigmaPlot version 10 software (Systat Software, Point Richmond, CA, USA).

## Electronic supplementary material

SI

## References

[1] Hardeland R, Madrid JA, Tan DX, Reiter RJ (2012). Melatonin, the circadian multioscillator system and health: the need for detailed analyses of peripheral melatonin signaling. Journal of pineal research.

[2] He C (2016). Melatonin and its receptor MT1 are involved in the downstream reaction to luteinizing hormone and participate in the regulation of luteinization in different species. Journal of pineal research.

[3] He C (2016). Mitochondria synthesize melatonin to ameliorate Its function and improve mice oocyte’s quality under *in vitro* conditions. International Journal of Molecular Sciences.

[4] He C (2015). Melatonin-related genes expressed in the mouse uterus during early gestation promote embryo implantation. Journal of pineal research.

[5] Bizzarri M, Proietti S, Cucina A, Reiter RJ (2013). Molecular mechanisms of the pro-apoptotic actions of melatonin in cancer: a review. Expert opinion on therapeutic targets.

[6] Balzer I, Hardeland R (1991). Photoperiodism and effects of indoleamines in a unicellular alga. Gonyaulax polyedra. Science.

[7] Dubbels R (1995). Melatonin in edible plants identified by radioimmunoassay and by high performance liquid chromatography-mass spectrometry. Journal of pineal research.

[8] Hernández-Ruiz, J. & Arnao, M. B. Phytomelatonin, an interesting tool for agricultural crops. *Focus Sci* **2** (2016).

[9] Arnao, M. B. P: discovery, content, and role in plants. *Advances in Botany* **2014** (2014).

[10] Ebihara S, Marks T, Hudson DJ, Menaker M (1986). Genetic control of melatonin synthesis in the pineal gland of the mouse. Science.

[11] Back, K., Tan, D. X. & Reiter, R. J. Melatonin biosynthesis in plants: multiple pathways catalyze tryptophan to melatonin in the cytoplasm or chloroplasts. *Journal of Pineal Research* (2016). 10.1111/jpi.12364 27600803

[12] Arnao, M., Hernández-Ruiz, J. & DeMello, J. Melatonin: synthesis from tryptophan and its role in higher plants. *Amino acids in higher plants: CAB eBooks*, 390-435 (2015).

[13] Nawaz, M. A. *et al*. Melatonin: current status and future perspectives in plant science. *Frontiers in plant science* **6** (2015). 10.3389/fpls.2015.01230 PMC4707265 26793210

[14] Okazaki M, Higuchi K, Aouini A, Ezura H (2010). Lowering intercellular melatonin levels by transgenic analysis of indoleamine 2, 3-dioxygenase from rice in tomato plants. Journal of pineal research.

[15] Hernández-Ruiz J, Arnao MB (2008). Melatonin stimulates the expansion of etiolated lupin cotyledons. Plant Growth Regulation.

[16] Arnao MB, Hernández-Ruiz J (2015). Functions of melatonin in plants: a review. Journal of pineal research.

[17] Arnao MB, Hernández-Ruiz J (2014). Melatonin: plant growth regulator and/or biostimulator during stress?. Trends in plant science.

[18] Shi, H., Chen, K., Wei, Y. & He, C. Fundamental issues of melatonin-mediated stress signaling in plants. *Frontiers in Plant Science* **7** (2016). 10.3389/fpls.2016.01124 PMC4961697 27512404

[19] Zhang N (2015). Roles of melatonin in abiotic stress resistance in plants. Journal of Experimental Botany.

[20] Hernández-Ruiz J, Cano A, Arnao MB (2005). Melatonin acts as a growth-stimulating compound in some monocot species. Journal of pineal research.

[21] Arnao M, Hernández-Ruiz J (2009). Protective effect of melatonin against chlorophyll degradation during the senescence of barley leaves. Journal of pineal research.

[22] Byeon Y, Back K (2014). An increase in melatonin in transgenic rice causes pleiotropic phenotypes, including enhanced seedling growth, delayed flowering, and low grain yield. Journal of pineal research.

[23] Zhao Y (2013). Melatonin and its potential biological functions in the fruits of sweet cherry. Journal of Pineal Research.

[24] Fu C (2011). Genetic manipulation of lignin reduces recalcitrance and improves ethanol production from switchgrass. Proceedings of the National Academy of Sciences.

[25] Kang K, Lee K, Park S, Kim YS, Back K (2010). Enhanced production of melatonin by ectopic overexpression of human serotonin N-acetyltransferase plays a role in cold resistance in transgenic rice seedlings. Journal of pineal research.

[26] Wang L (2014). Changes in melatonin levels in transgenic ‘Micro-Tom’tomato overexpressing ovine *AANAT* and ovine *HIOMT* genes. Journal of Pineal Research.

[27] Cai, S. Y. *et al*. HsfA1a upregulates melatonin biosynthesis to confer cadmium tolerance in tomato plants. *Journal of Pineal Research* **62** (2017). 10.1111/jpi.12387 28095626

[28] Xu W (2016). Melatonin enhances thermotolerance by promoting cellular protein protection in tomato plants. Journal of Pineal Research.

[29] Park S (2013). Melatonin‐rich transgenic rice plants exhibit resistance to herbicide-induced oxidative stress. Journal of Pineal Research.

[30] Chen G (2003). Melatonin in Chinese medicinal herbs. Life sciences.

[31] Ramakrishna A, Giridhar P, Sankar KU, Ravishankar GA (2012). Melatonin and serotonin profiles in beans of Coffea species. Journal of pineal research.

[32] Okazaki M, Ezura H (2009). Profiling of melatonin in the model tomato (*Solanum lycopersicum* L.) cultivar Micro-Tom. Journal of pineal research.

[33] Van Tassel DL, Roberts N, Lewy A, O’neill SD (2001). Melatonin in plant organs. Journal of pineal research.

[34] Murch, S. J., KrishnaRaj, S. & Saxena, P. K. in *Transplant Production in the 21st Century*, 160–165 (Springer, 2000).

[35] Kolář J (1997). Melatonin: occurrence and daily rhythm in *Chenopodium rubrum*. Phytochemistry.

[36] Afreen F, Zobayed S, Kozai T (2006). Melatonin in Glycyrrhiza uralensis: response of plant roots to spectral quality of light and UV-B radiation. Journal of pineal research.

[37] Stehle JH, von Gall C, Schomerus C, Korf H-W (2001). Of rodents and ungulates and melatonin: creating a uniform code for darkness by different signaling mechanisms. Journal of Biological Rhythms.

[38] Hernandez-Ruiz J, Cano A, Arnao MB (2004). Melatonin: a growth-stimulating compound present in lupin tissues. Planta.

[39] Chen Q, Qi W-b, Reiter RJ, Wei W, Wang B-m (2009). Exogenously applied melatonin stimulates root growth and raises endogenous indoleacetic acid in roots of etiolated seedlings of Brassica juncea. Journal of plant physiology.

[40] Kolář J, Johnson CH, Macháčková I (2003). Exogenously applied melatonin (N-acetyl-5-methoxytryptamine) affects flowering of the short-day plant Chenopodium rubrum. Physiologia Plantarum.

[41] Arnao MB, Hernández-Ruiz J (2006). The physiological function of melatonin in plants. Plant signaling & behavior.

[42] Shi H (2015). Comparative physiological, metabolomic, and transcriptomic analyses reveal mechanisms of improved abiotic stress resistance in bermudagrass [*Cynodon dactylon* (L). Pers.] by exogenous melatonin. Journal of experimental botany.

[43] Li C (2012). The mitigation effects of exogenous melatonin on salinity-induced stress in *Malus hupehensis*. Journal of pineal research.

[44] Tal O, Haim A, Harel O, Gerchman Y (2011). Melatonin as an antioxidant and its semi-lunar rhythm in green macroalga *ulva sp*. Journal of experimental botany.

[45] Wang P (2012). Delayed senescence of apple leaves by exogenous melatonin treatment: toward regulating the ascorbate–glutathione cycle. Journal of pineal research.

[46] Liu Y-R, Cen H-F, Yan J-P, Zhang Y-W, Zhang W-J (2015). Inside out: high-efficiency plant regeneration and *Agrobacterium*-mediated transformation of upland and lowland switchgrass cultivars. Plant cell reports.

[47] Li J-l, Wang S, Jing Y, Wang L, Zhou S-l (2013). A modified CTAB protocol for plant DNA extraction. Chinese Bulletin of Botany.

[48] Hardin CF (2013). Standardization of switchgrass sample collection for cell wall and biomass trait analysis. BioEnergy Research.

[49] Livak KJ, Schmittgen TD (2001). Analysis of relative gene expression data using real-time quantitative PCR and the 2^−ΔΔCT^ method. methods.

[50] Niu L (2016). *Control of floral transition* in the bioenergy crop switchgrass. Plant, cell & environment.

[51] Paudel, B. *et al*. Proteomic responses of switchgrass and prairie cordgrass to senescence. *Frontiers in plant science* **7** (2016). 10.3389/fpls.2016.00293 PMC4789367 27014316

[52] Bao A-K (2009). Overexpression of the Arabidopsis H^+^-PPase enhanced resistance to salt and drought stress in transgenic alfalfa (*Medicago sativa* L. Plant Science.

[53] Lutts S, Kinet J, Bouharmont J (1996). NaCl-induced senescence in leaves of rice (*Oryza sativa* L.) cultivars differing in salinity resistance. Annals of Botany.

[54] Peever TL, Higgins VJ (1989). Electrolyte leakage, lipoxygenase, and lipid peroxidation induced in tomato leaf tissue by specific and nonspecific elicitors from *Cladosporium fulvum*. Plant Physiology.

[55] Bates L, Waldren R, Teare I (1973). Rapid determination of free proline for water-stress studies. Plant and soil.

[56] Hartmut K (1983). Determinations of total carotenoids and chlorophylls b of leaf extracts in different solvents. Biochemical Society Transactions.

[57] Wang B, Zhao K (1996). Changes in Na and Ca concentrations in the apoplast and symplast of the etiolated corn seedlings under NaCl stress. Acta Agronomica Sinica.

