# Supplementary material for: Overexpression of ovine AANAT and HIOMT genes in switchgrass leads to improved growth performance and salt-tolerance
Source: Sci Rep. 2017 Sep 22;7:12212. doi: 10.1038/s41598-017-12566-2 (PMC5610178; doi:10.1038/s41598-017-12566-2)
Supplement: Supplementary file 1 — SI [file 41598_2017_12566_MOESM1_ESM.docx]

Article title: **Overexpression of ovine *AANAT* and *HIOMT* genes in switchgrass leads to improved growth performance and salt-tolerance**

Authors: Yan-Hua Huang ^1,2^, Si-Jia Liu^1^, Shan Yuan^1^, Cong Guan^1^, Dan-Yang Tian^1^, Xin Cui^1^, Yun-Wei Zhang ^1,3,4*^ and Fu-Yu Yang ^1,5*^

**The following Supporting Information is available for this article:**

**Figure S1** The plant heights of transgenic switchgrass and correlation analysis of plant heights and melatonin levels. (a) and (b): Plant height in different OE-*oAANAT* and OE-*oHIOMT* lines; (c) and (d): Correlation analysis of melatonin levels and plant heights of *oAANAT* and *oHIOMT*, respectively. WT: wild type; EV: lines expressing the empty vector only; The data show the mean ± S.E. of triplicate measurements. * and ** indicate a significant difference from that of WT at *P* < 0.05 and *P* < 0.01, respectively.


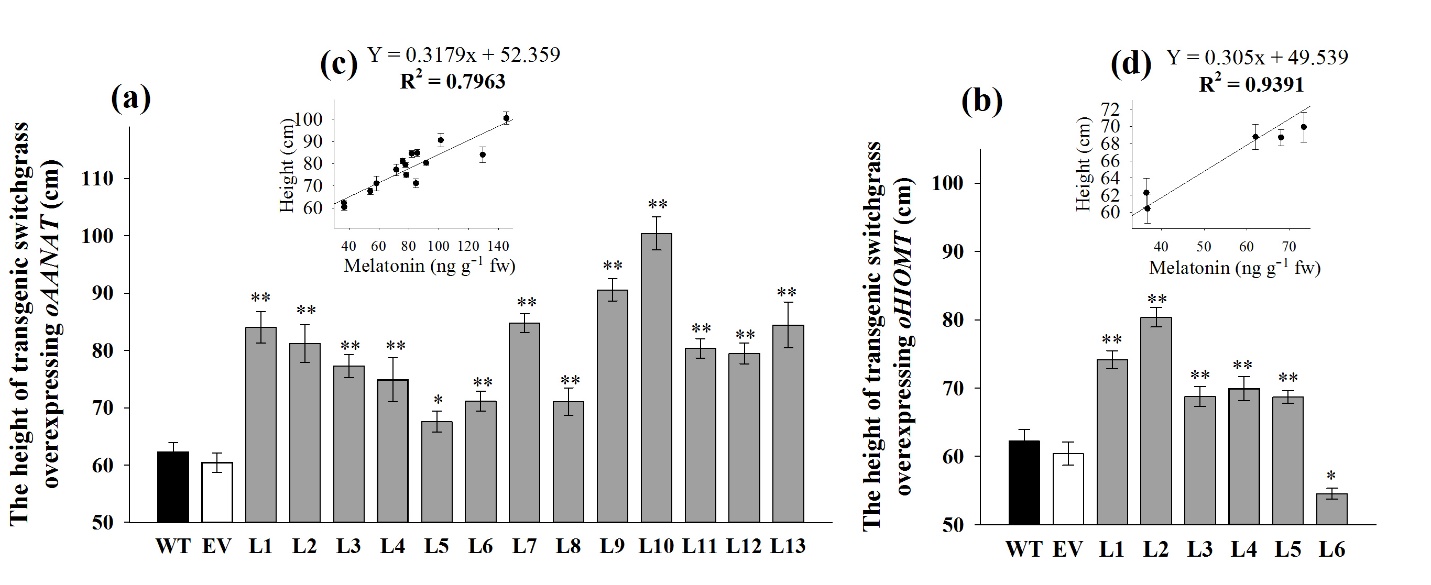


**Figure S2** Physiological analyses of WT and transgenic switchgrass lines treated with 400 mM NaCl solution for 0, 10, 20, 30 and 40 d, respectively. The variation tendency of RWC (a), EL (b), proline (c) and MDA content (d) under different treatment time; Average variation tendency of RWC (e), EL (f), proline (g) and MDA (h); WT: wild type; A1, A9 and A10: the transgenic plants overexpressing *oAANAT*; H1, H2 and H6: the transgenic plants overexpressing *oHIOMT*. The data show the mean ± S.E of three plicate samples.


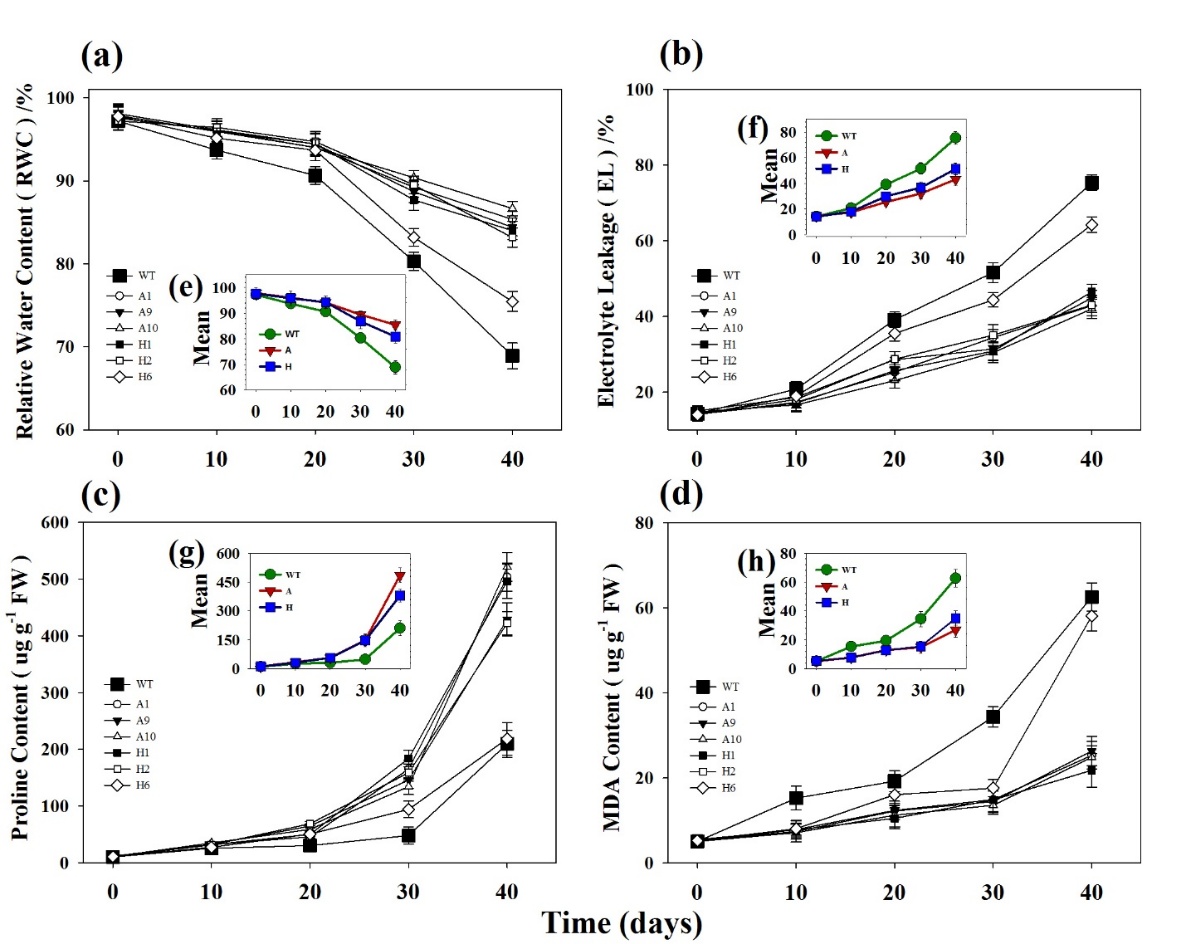


**Figure S3** Total chlorophyll content in WT and transgenic switchgrass under different concentrations of salt stress. (a) Total chlorophyll content in OE-*oAANAT* transgenic lines; (b) Total chlorophyll content in OE-*oHIOMT* transgenic lines. The data represents the mean ± S.E of three replicate samples. Statistically significant values at *P* < 0.05 using ANOVA analysis are indicated by different letters.

**
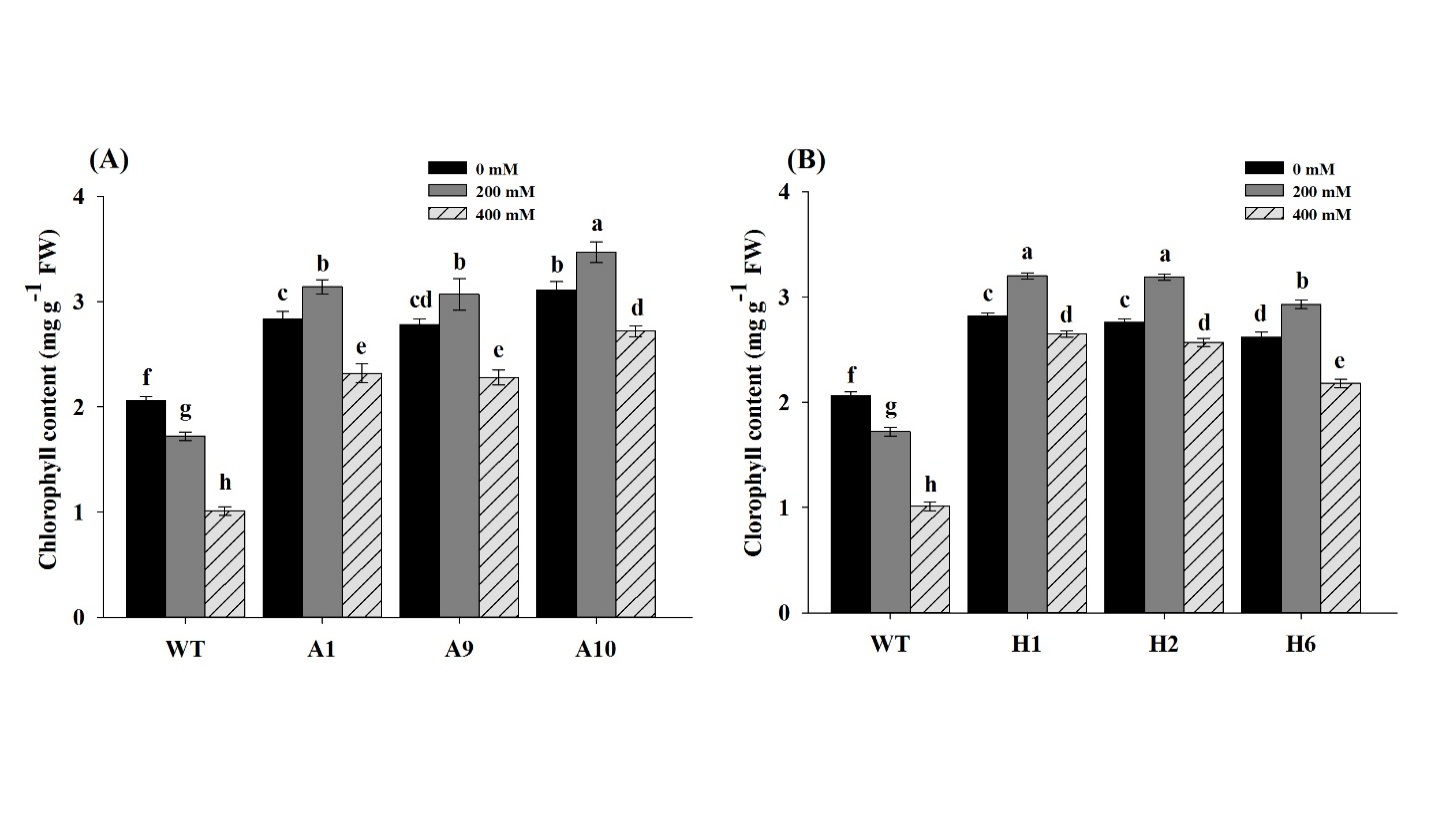
**

**Figure S4** K^+^/Na^+^ ratios in tissues from WT and transgenic switchgrass lines under different concentrations of salt stress. (a) K^+^/Na^+^ in shoots; (c) K^+^/Na^+^ in roots; (b) Average ratio of K^+^/Na^+^ in shoots; (d) Average ratio of K^+^/Na^+^ in roots; The data show the mean ± S.E of three plicate samples.

**
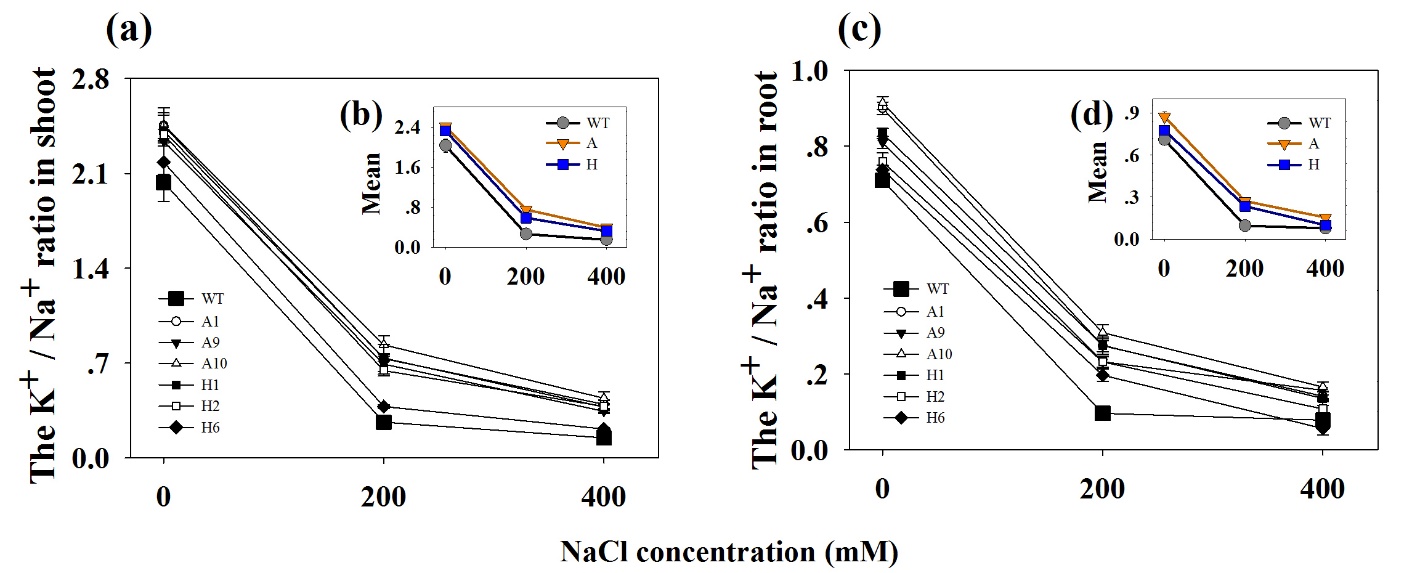
**

**Figure S5** Effects of salt stress on the expression of *PvNHX1* in transgenic and WT plants. Expression analysis of *PvNHX1* in OE-*oAANAT* transgenic lines (a) and *PvNHX1* in OE-*oHIOMT* transgenic lines (b). The data represents the mean ± S.E of three replicate samples. Statistically significant values at *P*< 0.05 using ANOVA analysis are indicated by different letters.

**
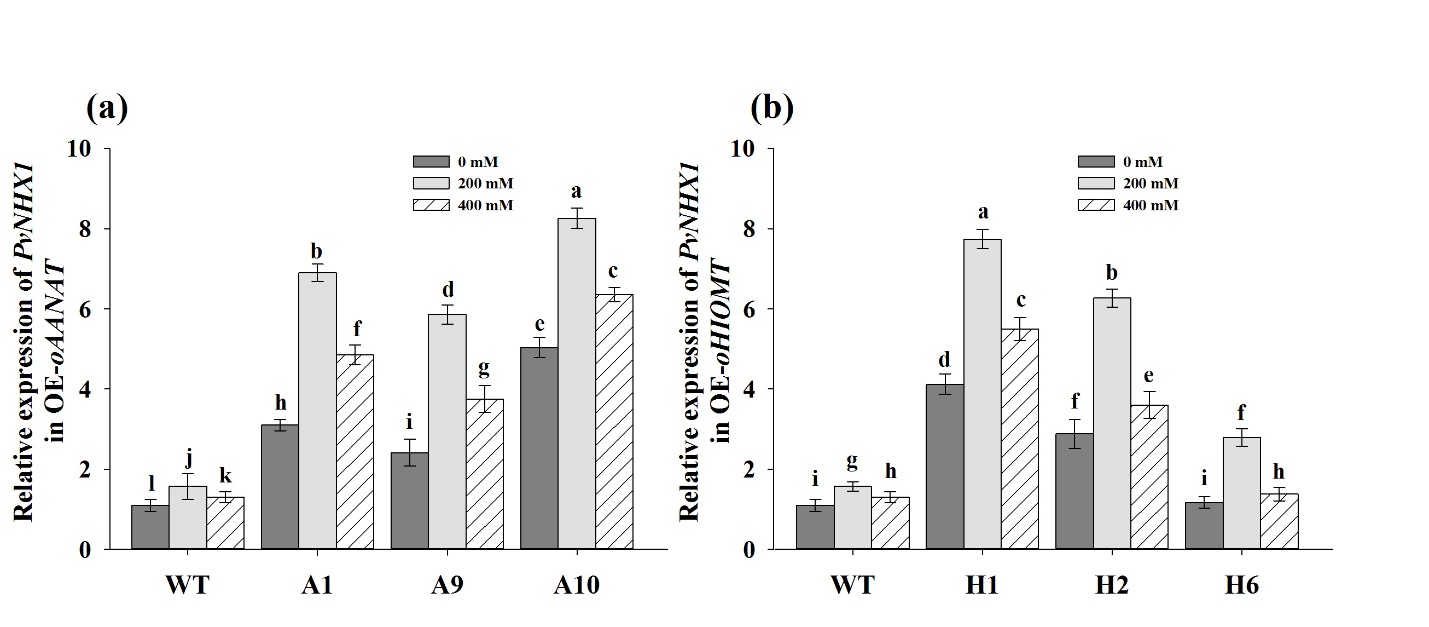
**

**Figure S6** The full-length gels of PCR and southern analysis in transgenic plants. PCR analysis of transgenic plants overexpressing *oAANAT* (a) and *oHIOMT* (b). M: DL 2000 molecular weight marker; +: plasmid (positive control); -: wild type and H_2_O; Southern blot analysis of transgenic plants overexpressing *oAANAT* (c) and *oHIOMT* (d). M: 1 kb plus DNA ladder; +: plasmid (positive control); -: wild type (negative control).


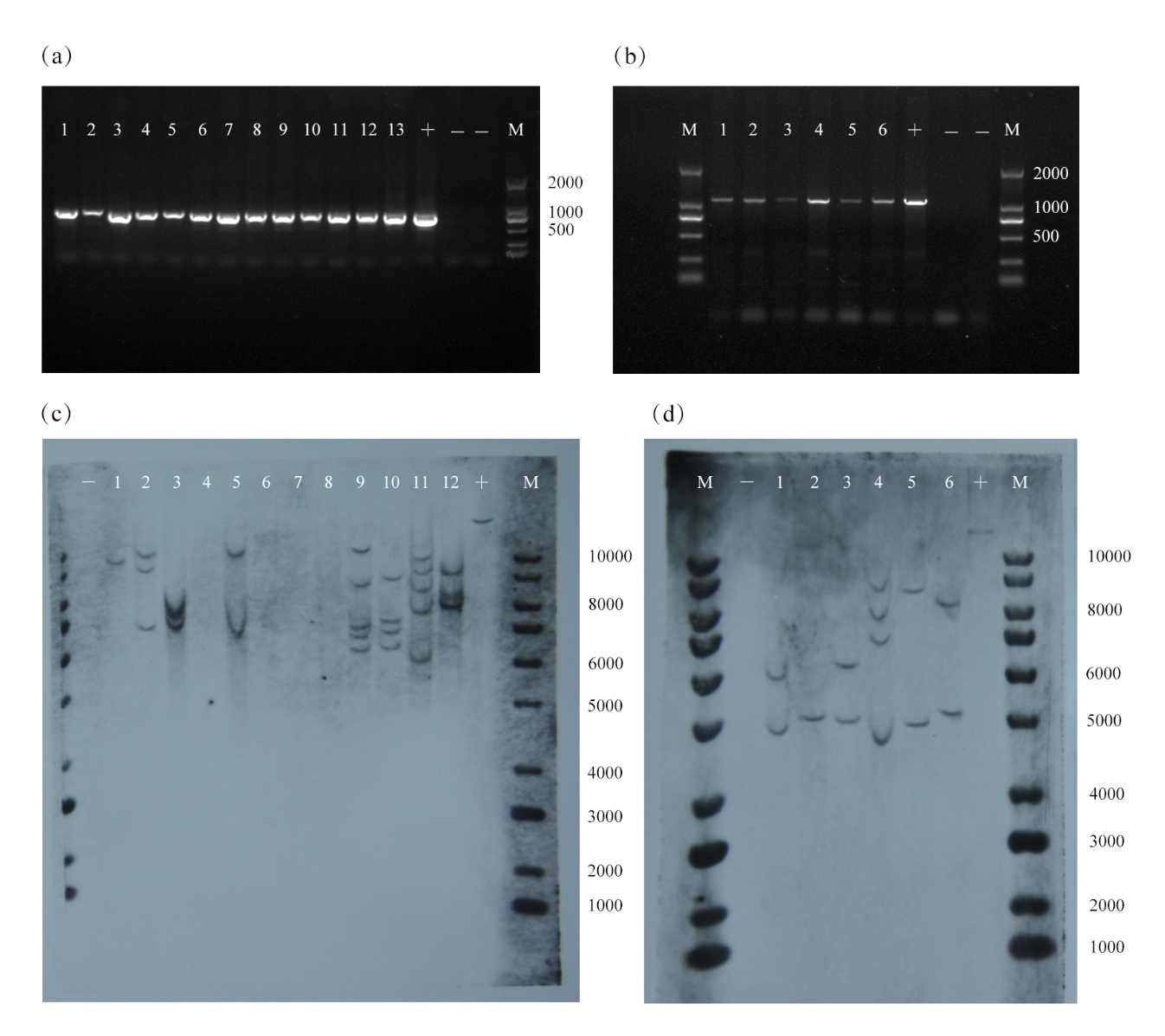


**Table S1** Primer sequences used in the experiments.

| **Primer name** | **Primer sequence (5'-3')** |
| --- | --- |
| *oAANAT*–F1 | CCACCATGTCCACGCCGAGC |
| *oAANAT*–R1 | GTCAGCGGTCACTGTTCC |
| *oHIOMT*–F1 | CCACCATGTGCTCCCAGGAG |
| *oHIOMT*–R1 | CCTCACTTT CTGGCCAAGA |
| U*b*i1301-F | TGATGGCCCTGCCTTCATACGCT |
| Ubi1301-R | TATTGCCAAATGTTTGAACG |
| hyg-F | ATTTGTGTACGCCCGACAGT |
| hyg-R | GGATATGTCCTGCGGGTAA A |
| *oAANAT*–F2 | CACTTCCTGACCCTGTGTCC |
| *oAANAT*–R2 | GCGA CTCCTGAGTAAGTCTCTC |
| *oHIOMT*–F2 | AGGACGAGCGGATCAGCTTCC |
| *oHIOMT*–R2 | AGCACTTGGCATCGGTCCAGTC |
| *PvUBQ1*-F | CAGCGAGGGCTCAATAATTCCA |
| *PvUBQ1*-R | TCTGGCGGACTACAATATCCA |
| *PvNHX1*-F | TTGTGCTCTTCAATGCGCTT |
| *PvNHX1*-R | GCAATCCAGCAAACACTCCA |
| *FT1*-F | CTCAGGGAGTACTTGCACTG |
| *FT1*-R | TTGGCAGTTGAAGTAGACGG |
| *PvAPL3*-F | GAACCTGAATCGTCTCCTGC |
| *PvAPL3*-R | GGGTGTTGGTGACGGTGT |
| *PvSL1*-F | GAAGGAGAAGGAGATGACACTG |
| *PvSL1*-R | GACTTCTGCAACCACGTTTC |
| *FLP3*-F | AGCGCTACGTGAACGAC |
| *FLP3*-R | GAGCCTGAACCTGGCGAA |
| *MADS15*-F | AATTGGTGCCATGAATACAGGA |
| *MADS15*-R | TCAGAGACTCTAGATCCTCTCCCA |
| *MADS14*-F | ACGGTATGAACGCTACTCCT |
| *MADS14*-R | TCTCAACCTTCGCCTTTAGCA |
